# Virtual Reality Treatment for Public Speaking Anxiety in Students. Advancements and Results in Personalized Medicine

**DOI:** 10.3390/jpm10010014

**Published:** 2020-03-01

**Authors:** Francisco-Javier Hinojo-Lucena, Inmaculada Aznar-Díaz, María-Pilar Cáceres-Reche, Juan-Manuel Trujillo-Torres, José-María Romero-Rodríguez

**Affiliations:** Department of Didactics and School Organization, University of Granada, 18071 Granada, Spain; fhinojo@ugr.es (F.-J.H.-L.); iaznar@ugr.es (I.A.-D.); caceres@ugr.es (M.-P.C.-R.); jttorres@ugr.es (J.-M.T.-T.)

**Keywords:** virtual reality, anxiety disorders, public speaking anxiety, Internet-based interventions, personalized medicine

## Abstract

Public speaking anxiety (PSA) is a common phobia in the student population. Traditionally, exposure therapy has been used as a treatment. However, the use of virtual reality (VR) is increasingly common to treat PSA. The purpose of this paper was to analyze the published scientific literature on VR as a treatment for PSA in students. The articles indexed in two databases (Web of Science and Scopus) were analyzed, with a time period from the beginning of the first publications until 2019 included. The systematic literature review was based on fixed inclusion and exclusion criteria. A total of 13 studies were identified which included 481 students. The results collected indicate that the duration of treatments to have positive effects was at least one week, where the number of sessions was between one and twelve. Furthermore, most VR treatments reported positive effects. Finally, this study showed evidence that VR treatment for PSA is effective while being less invasive than in vivo exposure.

## 1. Introduction

Social anxiety disorder (SAD) is one of the most common disorders affecting the population [1]. This relates to the fear of being observed and judged by others [2]. Within SAD, the most common social phobia is public speaking anxiety [3]. Thus, most people diagnosed with SAD report that they are afraid to speak in public [4]. Furthermore, public speaking anxiety (PSA) is related to the fear of speaking in front of others, which increases the anxiety levels of the person in a public speaking situation [5]. SAD detection is usually performed with standardized self-report scales, where respondents assess their level of anxiety in certain situations [6].

In recent years, cases of PSA have been common in the student population [7,8]. Students begin to present worrying symptoms in relation to the increase in PSA. This is a problem if we consider that public speaking is one of the most important skills in society, even more so when it comes to professions related to the fields of education, health, economic sciences, or legal sciences. What is also of concern is the increase of this type of disorder in the new generations, encouraged by the social isolation that is being aggravated by the use of social networks and Internet addiction [9,10]. Not interacting face-to-face is becoming an aggravating factor in PES and this is favored by the excessive time spent by young people on the network [11]. Therefore, analyzing PSA in the student population is important to know how this disorder is evolving with the current development of technology.

Cognitive therapy (CT) has traditionally been used to treat PSA [12,13], as has cognitive behavioral therapy (CBT), where the patient is gradually exposed to feared social situations [1]. Therefore, the most commonly used technique in anxiety disorders is exposure therapy. However, it is increasingly common to develop experimental virtual reality (VR) therapies. 

Exposure therapy combined with VR allows the user to face a digital version of the feared situation, rather than a real situation [14]. It also overcomes the limitations of CBT related to the difficulty the patient may have in imagining the object of fear (imaginary exposure) or creating a controlled environment (in vivo exposure) [15]. All this has led to the rise of virtual reality exposure therapy (VRET) for the treatment of PSA [16], sometimes combined with cognitive behavioral therapy (VR-CBT) [17]. Thus, VR has been increasingly used in the treatment of anxiety disorders [18]. Specifically, VR allows to generate three-dimensional situations that are generated by computer. This allows users to experience situations similar to those in the real world [19], such as facing an oral presentation in an auditorium or virtual classroom [20,21]. In addition, 360º recording technology generates virtual environments with greater realism [8]. These environments are so real that some studies claim that virtual spectators in a classroom can affect people’s beliefs, anxiety, and behavior [7,22,23]. Unlike in vivo exposure techniques, VR generates virtual environments that favor privacy, avoid public embarrassment, and maintain patient confidentiality [24]. On the other hand, some studies have obtained results confirming that exposure to VR is as effective as exposure to group therapy [25,26,27,28]. In turn, VR has begun to be used with students, where experiences show that it increases motivation and especially helps to reduce anxiety about exams and real situations [29,30,31].

The inclusion of VR as a treatment for anxiety has been addressed in a number of previous systematic reviews and/or meta-analysis studies. All of them have different approaches and objectives:

(i) Analysis of VR exposure attrition rate and in vivo exposure therapy. The review collected 46 studies. The meta-analysis obtained concise data that both therapies show similar attrition rates [32].
(ii) Applicability of VR to mental health disorders. Twenty-nine studies were identified that applied VR for anxiety disorders, depression, schizophrenia, psychosis, eating disorders, and obsessive-compulsive disorder [33].
(iii) Effectiveness of VRET for social anxiety. The meta-analysis results of the six studies analyzed showed a significant overall effect size. Thus, VRET was effective in reducing social anxiety [34].
(iv) Analysis of VR interventions for anxiety, depression, and treatment wasting outcomes. Meta-analysis results showed that VR interventions overcame control conditions for anxiety and depression, but did not improve desertion from treatment [35].
(v) Applicability of VR to health problems. Fifty studies were analyzed where different VR uses were detected to treat problems related to fear of flying, PSA, arachnophobia, agoraphobia, body image disturbance, and obesity [36].
(vi) Effectiveness of VRET for the treatment of anxiety disorders. The analysis found that VRET is effective for the treatment of anxiety, phobias, panic disorder, and post-traumatic stress disorder [37].
(vii) Effectiveness of VRET for anxiety-related disorders. Forty-nine studies were included where the majority reported positive findings in favor of VRET [38].
(viii) Comparison of the efficacy of VRET with classic treatments for anxiety. The 23 studies identified concluded in the meta-analysis that VRET is equally as effective as classical treatments, with no differences in dropout rate or stability of results over time [39].
(ix) Reduction of anxiety of different types (social phobia, arachnophobia, agoraphobia, acrophobia, aviophobia) through the VRET. Twenty-one studies were reviewed that found that anxiety symptoms were diminished with VR treatment [40].
(x) Measurement of the size of the effect of VR for the treatment of anxiety disorders. The meta-analysis collected a large average effect size for VRET compared with in vivo exposure and control conditions [41].

In short, previous revisions have analyzed studies that collect a general population, not based on a specific population such as students. In addition, PSA has not been addressed as a main topic. Therefore, prior to this study there is no systematic review or meta-analysis on the efficacy of VRET for the treatment of PSA in students. This led to the establishment of the following objective: (i) to analyze the published scientific literature on VR as a treatment for PSA in students.

The research questions were as follows:

- RQ1. How many studies were published over the years?
- RQ2. Who are the most active authors in the area?
- RQ3. In which sources appear this kind of study?
- RQ4. What is the educational stage and the country of the students?
- RQ5. How long was the treatment and what sequence was used?

## 2. Methods

This study used a systematic review methodology [42,43]. This type of analysis allows, on the one hand, the investigation of a specific topic and, on the other hand, the global effect of research on this topic. Furthermore, this method is widely consolidated and applied in Health Sciences. According to Marín, Sánchez, and López (2009) [44] (p. 107), this method allows us to analyze "the quantitative results of empirical studies on the same health problem, allowing health professionals to make well-informed decisions in their respective areas of work". In this paper, the topic was VR as a treatment for PSA in students. The Preferred Reporting Items for Systematic Reviews and Meta-Analyses (PRISMA) protocol [45] was applied.

The analysis variables collected were typical of a systematic review [46]. They answered the research questions raised. Thus, information regarding years of publication, authors, source title, characteristics of the students, and VR treatment was analyzed. 

### 2.1. Search Strategy

The search was established from the application of different descriptors in the Web of Science (WoS) and SCOPUS databases. These two databases have internationally recognized impact indices: Journal Citation Reports (JCR) in WoS and Scimago Journal & Country Rank (SJR) in SCOPUS [47].

The descriptors were applied in the search engine of both databases to proceed to further refining through the inclusion and exclusion criteria (Table 1). The search equation was defined around the key descriptors most commonly used in the literature on VR as a treatment for PSA [1,12,13] (Table 2).

To avoid bias in study selection, the systematic review was undertaken by three investigators (PhD in Education Sciences). All applied the same descriptors and inclusion/exclusion criteria. Although there was some discrepancy in the first round of search, in the second round the degree of agreement was 100%. The disagreement was between two investigators and was resolved by the third.

### 2.2. Data Collection and Analysis

**Figure 1:**
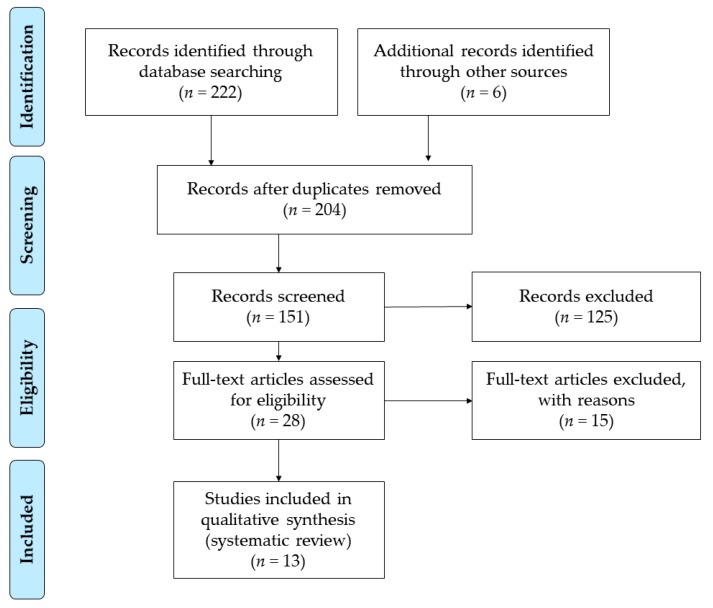
Flow diagram.

The literature screening process followed the four phases of the PRISMA protocol [45] (Figure 1). The identification phase consisted of an initial search of the WoS and SCOPUS databases and the identification of other documents from external sources. In this phase, duplicate references were eliminated. In the screening phase, the inclusion (IC1, IC2, IC3) and exclusion (EX1, EX2, EX3) criteria were applied. Subsequently, in the eligibility phase, each title and summary of articles were analyzed according to the inclusion (IC4, IC5) and exclusion (EX4, EX5) criteria. Finally, in the included phase, the final analysis sample was determined for the systematic review (*n* = 13) [48,49,50,51,52,53,54,55,56,57,58,59,60]. The search was conducted on 5 January, 2020. All articles published to date were reviewed.

The analysis strategy used for the systematic review was comprehensive reading [43]. Based on this, key information was extracted from each analysis variable. 

## 3. Results

### 3.1. RQ1. How Many Studies were Published over the Years?

**Figure 2:**
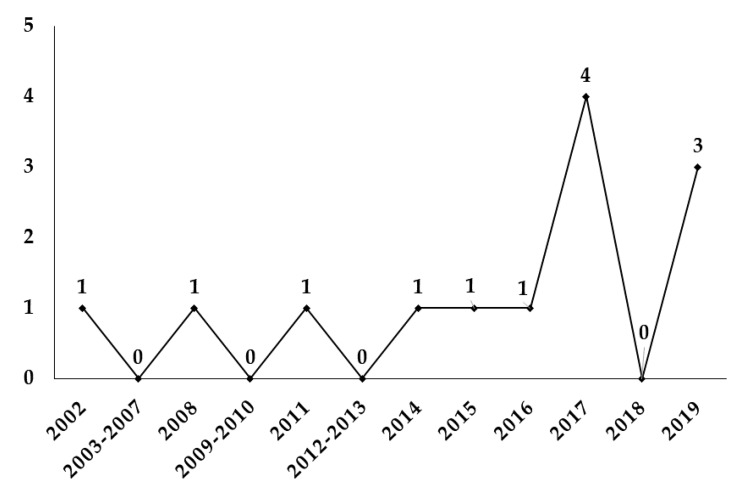
Number of articles per year.

The scientific literature on VR as a treatment for PSA in students began in 1997 [24], although the first empirical publication was in 2002. Since that year, it has had an irregular growth until 2014. In the same year, a period of stabilization began, reaching the peak of production in 2017, with four publications (30.76%). Subsequently, the irregularity has continued, with 0 publications in 2018 and a rebound in 2019. In general, the number of articles per year has been minimal during the years 2002–2016 and its growth has been irregular, marked by two periods where publications have rebounded (2017 and 2019) (Figure 2). 

### 3.2. RQ2. Who are the Most Active Authors in the Area?

As to the authorship of the articles, most were signed by several authors. However, most of the authors have only signed one article on this topic. Only three authors submit two articles and only one author appears in three articles (Table 3).

### 3.3. RQ3. In Which Sources Appear this Kind of Studies?

On the other hand, a total of 10 journals published articles on VR as a treatment for PSA in students (Table 4). Annual Review of Cybertherapy and Telemedicine collected three papers (23.07%) and Cyberpsychology, Behavior, and Social *Networking* presents two papers (15.38%). The rest only included an article on the topic. In addition, the greatest impact, based on the *h*-index, was presented by Cyberpsychology, Behavior, and Social Networking (*h*-index = 50).

### 3.4. RQ4. What is the Educational Stage and the Country of the Students?

The sample size of the studies analyzed ranged from 14 to 80 students. The total was 481. As for the characteristics of the students, almost all the studies had university students as a sample (84.61%). Only two papers focused on the High School stage [56]. The average age of the students was between 16 and 31.36 years old (*M* = 22.67; *SD* = 4.82). The countries of origin were USA (23.07%), Germany (23.07%), Netherlands (15.38%), Turkey (7.69%), UK (7.69%), Norway (7.69%), Sweden (7.69%), and Georgia (7.69%) (Table 5).

### 3.5. RQ5. How Long was the Treatment and what Sequence was Used?

With regard to treatment characteristics (Table 6), in all studies, only VRET was applied as a treatment. Therefore, it was not combined with any other technique. In only three of the cases were the students diagnosed with social anxiety disorder [48,52,56]. The rest showed symptoms but were not diagnosed. Treatment varied from study to study, ranging from one day to three months (*M* = 20.25 days). The number of treatment sessions was a minimum of one and a maximum of 12, with the shortest lasting five minutes and the longest 90 minutes (*M* = 19.76; *SD* = 23.26). In terms of group allocation, the majority were participant matching (53.84%). This type of study did not make a division between groups with VRET and control, and the treatment was applied to the whole group. The rest did divide into two distinct groups, with a quasi-random (23.07%) and random (23.07%) assignment. The content of the interventions was based on the exposure of real situations of public speech. The recreated spaces were a virtual classroom (46.15%), auditorium (38.46%), everyday situations (7.69%), meeting room (7.69%), wedding reception (7.69%), and party and presentation environment (7.69%). Regarding the effect of the interventions, most of them showed a positive effect of the VRET (76.92%), only three studies showed a zero effect (23.07%).

## 4. Discussion

The analysis of the scientific literature showed the application of the VR technique as a PSA treatment in 481 students. The effect of the studies reviewed checked the possible efficacy of VRET for the treatment of PSA. The applicability of VR as a treatment for PSA began in 2002. Since that year, there has been an inconsistency in the publications. However, in 2017, research increased, although in 2018 it decreased again. Therefore, VRET used for the treatment of PSA in students requires more empirical research, since the literature found is limited and inconsistent.

In relation to authorship, the publications include a diversity of authors, where the manuscripts present a multiple authorship. This is a common feature in the health branch. The exception is found in a single article that is signed by a single author (Poeschl, S.). In addition, this same author is shown as the author with the highest number of publications on the subject. In turn, the journal with the largest number of publications is the Annual Review of Cybertherapy and Telemedicine, which indicates some interest in the subject matter on the part of this journal.

On the other hand, the most studied population is university students, over other educational stages. This reflects the fact that the university environment is a focus of interest for PSA research [7]. Without forgetting that higher education is the stage that prepares students to exercise a profession. So addressing PSA at this stage is critical to generating qualified professionals to adequately develop a job that requires public speaking. Another finding is the countries that conduct more research on the application of VR for the treatment of PSA. USA, Germany, and Netherland stand out as the main countries interested in the topic applied to students.

As for the application of VR, only VRET was applied [16,18]. It was not combined with exposure therapy in the various studies reviewed. Thus, students were only exposed to VR for the treatment of PSA-related symptoms. Therefore, VR may be effective on its own. It should be noted that the sample of students tested showed PSA symptoms prior to treatment [8]. These were detected with standardized self-report scales [6].

The duration of the treatments to have positive effects was at least one day, with four sessions or a single session with an extended duration. It was more common to apply four sessions with an average duration of 19 minutes.

Based on the allocation of treatment to the sample, most did not allocate treatment randomly. In this regard, the only studies that could be generalized are those by Denizci et al. (2018) [48], Harris et al. (2002) [50], and Lindner et al. (2019) [53], which presented data in favor of VRET with a random assignment.

Among the virtual environments generated with VR, most researches recreated a virtual classroom and an auditorium with a fictitious audience [20,21]. In addition, other studies virtualized everyday situations, which were made possible by the realism of the interactions [22,23].

With regard to the limitations, the search for scientific documents in two databases (WoS and SCOPUS) is highlighted as a limitation of the study. In spite of this, the use of these databases is relevant, since the literature with the greatest scientific impact, indexed in the JCR and SJR indexes, has been analyzed. Another limitation was the possible bias of the researchers. To avoid this, a double-round system was used in which three independent researchers participated.

## 5. Conclusions

VRET is emerging as a method that may be effective for the treatment of PSA in students. The creation of virtual environments allows to put the student in a fictitious situation where he can have control over the environment and be able to develop without fear. This makes it easier for him to speak in public without increasing anxiety levels. 

In this paper, we analyzed the published scientific literature on VR as a treatment for PSA in students. It is therefore a method that a priori obtains favorable results in the same way as traditional exhibition methods. Added to this is the advantage of being able to generate a controlled environment, where the student feels comfortable and safe.

The results of this study may contribute to the selection of patterns for applying PSA treatments, as they show key aspects such as the duration of the treatments, number of sessions and minutes, type of assignment, and content of the virtual reality exposures. Thus, it is recommended that empirical research on VR for the treatment of PSA in students continues in order to have more examples and to put an end to the instability of the literature on this topic. On the other hand, in the literature consulted, there are different software programs used in VR as PSA treatment. It is interesting to point them out as part of future recommendations: RTT DeltaGen 12.2; VREAM™ Virtual Reality Development Software Package and Libraries (VRCreator™); CryEngine3 (Version PC v3.4.0 3696 freeSDK); Discreet 3D Studio Max 4; and Virtools.

Finally, the clinical use of VR may be beneficial for students, to which is added the educational use and the possibilities that this technology can allow in order to teach professional skills. The use of VR in students should continue to be applied not only for the PSA treatment, but to develop skills that will enable them for the labor market in the 21st century.

## Figures and Tables

**Table 1:** Inclusion and exclusion criteria.

Inclusion Criteria (IC)	Exclusion Criteria (EX)
IC1: Journal articles.	EX1: Proceedings of congresses, book chapters, books, or other types of non-peer-reviewed publications.
IC2: Empirical research.	EX2: Theoretical studies or revisions.
IC3: The papers are written in English language.	EX3: The papers are not described in English.
IC4: Using Virtual Reality as a treatment for public speaking anxiety.	EX4: Virtual Reality is not used as a treatment for public speaking anxiety.
IC5: Population are students.	EX5: The study population are not students.

**Table 2:** Search topics.

Database	Search Descriptors	Papers
WoS	TS = (“Virtual reality” AND anxiety AND “Public speaking”). Time period = Every year. Index = SCI-EXPANDED, SSCI, A&HCI, CPCI-S, CPCI-SSH, BKCI-S, BKCI-SSH, ESCI, CCR-EXPANDED, IC.	139
SCOPUS	TITLE-ABS-KEY ("virtual reality" OR VR) AND TITLE-ABS-KEY (anxiety) AND TITLE-ABS-KEY ("public speaking"). Time period = Every year.	83

**Table 3:** Authors’ names and number of publications.

Author	Total
Poeschl, S.	3
Doering, N., Lindner, P., North, M.M.	2
Aikhuele, A.S., Andersen, J., Andersson, G., Bordnick, P., Brinkman, W.P., Carlbring, P., Claske, P., Denizci, M., Dubiago, M., Duron, J.F., Emmelkamp, P.M.G., Fagernäs, S., Fullwood, C., Furmark, T., Gozcu, M.A., Gulec, U., Harris, S.R., Hartanto, D., Heuett, B.L., Heuett, K.B., Hill, J., Isler, V., Kahlon, S., Kampmann, I.L., Kemmerling, R.L., Ketelaars, L.E.H., Miloff, A., Morina, N., Nordgreen, T., North, S.M., O’Connor, R.V., Oxhandler, H.K., Parrish, D.E., Sigeman, M., Stupar-Rutenfrans, S., Swank, P., Van Gisbergen, M.S., Wilsdon, L., Yilmaz, A.E., Yilmaz, M.	1

**Table 4:** Source title and *h*-index.

Reference	Journal	*h-*Index
[49,58,60]	Annual Review of Cybertherapy and Telemedicine	8
[50,59]	Cyberpsychology, Behavior, and Social Networking	50
[57]	Frontiers in ICT	15
[48]	IET Software	13
[55]	International Journal of Emerging Technologies in Learning	19
[53]	International Journal of Humanities and Social Science	4
[56]	Research on Social Work Practice	27
[54]	Technology and Health Care	18
[51]	Child and Adolescent Psychiatry and Mental Health	26
[52]	Journal of Anxiety Disorders	47

Note: Hyphen (-) = not identified.

**Table 5:** Characteristics of the students.

Reference	Educational Stage		*n*	Age (*M*)	Country
	High School	University			
Denizci et al. (2019) [48]		X	14	21.36	Turkey
Dubiago et al. (2017) [49]		X	40	25.60	Germany
Harris et al. (2002) [50]		X	14	-	USA
Heuett and Heuett (2011) [51]		X	80	20	USA
Kahlon et al. (2019) [52]	X		27	14.22	Norway
Lindner et al. (2019) [53]		X	50	31.36	Sweden
Morina et al. (2015) [54]		X	34	22.3	Netherlands
North et al. (2008) [55]		X	30	-	Georgia
Parrish et al. (2016) [56]	X		41	16	USA
Poeschl (2017) [57]		X	36	26.42	Germany
Poeschl and Doering (2014) [58]		X	40	24	Germany
Stupar-Rutenfrans, et al. (2017) [59]		X	35	-	Netherlands
Wilsdon and Fullwood (2017) [60]		X	40	25.5	UK

Note: Hyphen (-) = not identified in text.

**Table 6:** Characteristics of the treatment with VRET.

No. of Reference	Social Anxiety Disorder Diagnosis	Time Pre-Post Used in Analysis	No. of Treatment Sessions (Minutes)	Assignment	Content Exposed	Favour Effect
[48]	Yes	4 weeks	4 (8–10’)	Random	Auditorium	VRET
[49]	No	-	1 (5–6’)	Participant matching	Virtual classroom	VRET
[50]	No	4 weeks	4 (15’)	Random	Auditorium	VRET
[51]	No	1 week	1 (10–20’)	Quasi-random	Auditorium	VRET
[52]	Yes	1 day	1 (90’)	Participant matching	Virtual classroom	VRET
[53]	No	4 weeks	4 (20–30’)	Random	Auditorium, Meeting room and wedding reception	VRET
[54]	No	12 weeks	12 (30’)	Quasi-random	Everyday situations	VRET
[55]	No	5 weeks	4 (40–45’)	Participant matching	Auditorium	VRET
[56]	Yes	1 day	4 (5’)	Quasi-random	Party and presentation environment	VRET
[57]	No	1 day	2 (5’)	Participant matching	Virtual classroom	No effect
[58]	No	1 day	2 (5’)	Participant matching	Virtual classroom	No effect
[59]	No	4 weeks	3 (5’)	Participant matching	Virtual classroom	VRET
[60]	No	1 day	1 (5’)	Participant matching	Virtual classroom	No effect

Note: Hyphen (-) = not identified in text; VRET = Virtual Reality Exposure Therapy.
